# Editorial: Diagnostic innovations for microbial pathogens in edible plants: cutting-edge technologies for enhanced detection

**DOI:** 10.3389/fmicb.2026.1777845

**Published:** 2026-01-27

**Authors:** Ravinder Kumar, Anirban Roy, Ying Zhai

**Affiliations:** 1 ICAR-Indian Agricultural Research Institute, New Delhi, India; 2 United States Department of Agriculture, Washington, DC, United States

**Keywords:** biosensors, edible crops, IoT in agriculture, plant pathogen diagnostics, portable field diagnostics

The continuous progression of microbial pathogens poses a major threat to the health and productivity of edible plants, with profound implications for agriculture and global food security. Pathogens including viruses, bacteria, fungi, and oomycetes remain among the most significant constraints to sustainable crop production worldwide, causing substantial yield and quality losses that undermine farmer livelihoods and international agricultural trade. These challenges are further aggravated by climate change, which is reshaping pathogen distributions and host susceptibility, and by intensified production systems that favor disease emergence and rapid spread (Kumar et al., 2023). In this rapidly changing agroecosystem, early, accurate, and deployable diagnostic tools have become an indispensable pillar of modern plant disease management. Traditional diagnostic methods, while reliable, often depend on complex workflows, specialized laboratory infrastructure, and extended turnaround times that limit their utility for timely decision-making in the field (Kumar et al., 2021). In response, recent technological advancements are driving a paradigm shift toward rapid, sensitive, and cost-effective diagnostic solutions tailored specifically for agricultural applications. The integration of Internet of Things (IoT) platforms, biosensors, chemical sensors, electrochemical CRISPR (E-CRISPR) systems, and spectral detection techniques represents a pioneering approach to enhancing pathogen detection in edible plants. Against this backdrop, the Research Topic “*Diagnostic Innovations for Microbial Pathogens in Edible Plants: Cutting-Edge Technologies for Enhanced Detection*” was conceived to showcase recent advances that move plant pathogen diagnostics beyond conventional laboratory-based assays toward portable, field-ready solutions. The four articles included in this Research Topic collectively demonstrate how innovations in molecular biology, assay design, and deployable technologies are reshaping the diagnostic landscape for microbial pathogens affecting edible crops. Together, they underscore both the significant progress achieved and the remaining challenges in translating diagnostic innovation into practical, scalable, and impactful solutions for sustainable agriculture.

## The need for next-generation diagnostics in edible crops

Accurate pathogen identification is fundamental to effective disease management. Many high-value vegetables, fruits, and staple food crops are affected by pathogens that establish asymptomatic or low-titer infections during early stages of disease development (Bukhamsin et al., 2025). In such cases, delayed detection can result in widespread pathogen dissemination before control measures are implemented. Consequently, there is a growing demand for diagnostic tools that are not only sensitive and specific but also rapid, affordable, and suitable for deployment at the point of need. The articles assembled in this Research Topic respond directly to these demands by advancing diagnostic strategies that prioritize operational simplicity while maintaining analytical rigor. Collectively, they underscore a paradigm shift in plant pathology from retrospective diagnosis toward real-time disease surveillance and proactive intervention.

## Molecular innovation: isothermal amplification and CRISPR-based diagnostics

A central theme emerging from this Research Topic is the increasing adoption of isothermal nucleic acid amplification technologies as alternatives to conventional PCR. Isothermal methods eliminate the need for thermal cycling, reducing equipment requirements and enabling faster amplification under field-compatible conditions. One of the most notable contributions in this Research Topic is the development of a recombinase polymerase amplification (RPA)-assisted CRISPR-Cas12a diagnostic platform for the detection of *Chili leaf curl virus* (ChiLCV), a major viral pathogen of chili and other solanaceous crops (Paul et al.). By targeting a highly conserved region of the viral genome, the assay achieves exceptional analytical sensitivity, detecting minute quantities of viral DNA with high specificity. Importantly, the CRISPR-based readout provides a clear and interpretable signal, enabling reliable discrimination between infected and non-infected samples. The successful application of this technology to field-collected samples and crude plant extracts demonstrates its potential as a scalable and adaptable platform for a wide range of microbial pathogens affecting edible crops.

## Addressing bacterial pathogens through simplified and robust assays

Accurate detection of bacterial pathogens is particularly challenging when infections are systemic, latent, or unevenly distributed within host tissues. The articles included in this Research Topic address these challenges by introducing robust molecular assays designed for reliable bacterial detection under practical conditions. One contribution focuses on the development and validation of a rapid, LAMP-based diagnostic method for *Xanthomonas albilineans*, the causal agent of sugarcane leaf scald (Chakraborty et al.). Although sugarcane is primarily an industrial crop, the methodological framework presented in this study has clear relevance for edible plants affected by xylem-colonizing bacterial pathogens. By combining reagent-free DNA release with isothermal amplification, the authors demonstrate a streamlined diagnostic workflow capable of detecting bacterial DNA directly from multiple plant tissues. The emphasis on validation using field samples further strengthens the relevance of this work for real-world applications.

## Toward portability and decentralization of plant diagnostics

A central theme of this Research Topic is the shift toward portable and decentralized plant pathogen diagnostics, bringing detection capabilities directly to the field and enabling faster, localized decision-making. Such technologies reduce dependence on centralized laboratories, minimize delays in sample transport, and enhance disease surveillance, particularly in resource-limited regions. Emphasis on robustness, ease of use, minimal sample processing, and clear result interpretation ensures that these tools are operationally viable under field conditions. Supporting this perspective, Yadav and Yadav highlight the growing importance of portable diagnostic platforms including handheld devices, smartphone-based systems, microfluidics, and lab-on-a-chip technologies—integrated with biosensors, nucleic acid amplification, and immunoassays. Their review underscores how coupling portable diagnostics with digital tools such as IoT, AI, and machine learning is transforming precision agriculture, while also noting ongoing challenges related to sensitivity, durability, and regulatory standardization.

## Implications for sustainable disease management and food security

The innovations highlighted in this Research Topic underscore the critical role of advanced diagnostics in promoting sustainable agriculture and global food security. Early and accurate pathogen detection enables precision disease management, reducing unnecessary chemical inputs, supporting integrated control strategies, and strengthening quarantine and certification systems. Collectively, these advances enhance the resilience of food production systems to climate change, emerging pathogens, and global trade pressures. Complementing these diagnostic advances, Dutta et al. demonstrated that the plant growth–promoting effects of VOCs emitted by Bacillus vallismortis (EXTN-1) are highly media-dependent, with sugar-rich media enhancing plant growth and nutrient-rich media causing growth inhibition due to ammonia toxicity. Their findings highlight how microbial metabolic outputs can be optimized to improve plant performance, reinforcing the broader importance of targeted, mechanism-informed innovations for sustainable crop production.

## Conclusion

In summary, the Research Topic “*Diagnostic Innovations for Microbial Pathogens in Edible Plants: Cutting-Edge Technologies for Enhanced Detection*” presents a timely and comprehensive snapshot of the evolving diagnostic landscape in plant pathology. The four articles included in this Research Topic demonstrate how innovative molecular tools, simplified workflows, and portable platforms are redefining pathogen detection in edible crops. Together, these contributions underscore the central role of diagnostics in sustainable plant disease management and highlight the potential of emerging technologies to transform how microbial pathogens are detected, monitored, and controlled.
